# Evaluation and comparison of intratumoural and intrastromal infiltrating lymphocytes with clinicopathological features in breast carcinoma patients who have received neoadjuvant chemotherapy - A cross-sectional study

**DOI:** 10.1016/j.amsu.2022.104308

**Published:** 2022-08-07

**Authors:** Rahul Rangan, Sujata R. Kanetkar, Suresh J. Bhosale, Satish V. Kakade, Nanda J. Patil, Anand Gudur

**Affiliations:** a Department of Pathology, Krishna Institute of Medical Sciences, Karad, Maharashtra, India; b Department of Surgery, Krishna Institute of Medical Sciences, Karad, Maharashtra, India; c Department of Community Medicine, Krishna Institute of Medical Sciences, Karad, Maharashtra, India; d Department of Oncology, Krishna Institute of Medical Sciences, Karad, Maharashtra, India

**Keywords:** Tumour-infiltrating lymphocytes, Neoadjuvant therapy, Breast neoplasms, Triple negative breast cancer, Treatment outcome, Prognosis, Female, Cross-sectional studies

## Abstract

**Background:**

The microenvironment of breast cancer plays a significant role in determining the prognosis of the disease. With the shifting paradigm on the predictive factors post-Neoadjuvant Chemotherapy (NAC), it was sought out that Tumour infiltrating lymphocytes (TILs) are of valuable use for the same. Yet, the delineation of the two types - Intrastromal and Intratumoural has seldom been facilitated. This study, therefore, aimed to evaluate, analyse and compare the two - to gauge the importance of the treatment outcome and clinicopathological features.

**Materials and methods:**

180 breast cancer patients were included in this study who underwent NAC, and their post-surgically resected tumour specimens were sectioned and stained using routine Haematoxylin and Eosin techniques. The evaluation of TILs in the stroma and tumour was done based on the standardised guidelines.

**Results:**

Out of the 180 patients, 55 (i.e. 30.56%) displayed pathological complete resolution (pCR). Furthermore, Intratumoural TILs had a slight association with the pCR (p = 0.0335) whereas Intrastromal TILs had a significantly large association with pCR (p < 0.0001) dependent on the lymphocytic response. Backward regression revealed that - the age at operation, pCR, lymph node involvement and menopause highly contributed to predicting 68.2% of the total cases correctly with a sensitivity of 93.0% and specificity of 24.6% for Intratumoral TILs. Age at operation, pCR, lymph node involvement and tumour emboli highly contributed to predicting 71.5% of the total cases correctly with sensitivity of 71.6% and specificity of 71.4% for Intrastromal TILs.

**Conclusion:**

TILs and the prediction of NAC and pCR should be made standardised and reproducible so that they can be universally available to all patients with breast cancer. Through this study, further avenues of research have opened up for their relations with clinicopathological features mainly age at operation and menopausal status.

## Highlights

• IT & IS TILs play a varied role in the prediction of NAC and pCR.
• Post-NAC 55 patients (i.e. 30.56%) out of 180 displayed pCR.
• Age at operation, pCR, lymph node involvement and menopause highly contributed to predict IT TILs.
• Age at operation, pCR, lymph node involvement and tumour emboli highly contributed to predict IS TILs.
• Analysis of TILs can be a reliable method in the treatment response and immunogenicity of breast tumours.

## 1 Introduction

Breast cancer is known for its heterogeneous histopathological, gross and immunohistochemical features. As mentioned by Walter E. Sistrunk and William C. MacCarty [1], “It is impossible to foretell the duration of life of all patients with carcinoma of the breast, because the degree of malignancy varies widely, and persons react differently to the disease”. Consequently, there are striking differences in the prognosis of breast cancer according to the tumour characteristics and immune response.

The body's immune system has proven to be significantly influential in the prognosis of breast cancer [2] and by observing this immunological response of the body to cancer in the microenvironment of the tumour we can find that the body plays a vital role in predicting the treatment response and outcome of the patient.

The varying immunohistochemical subtypes of breast cancer form the basis of multiple research modules specifically designed to assess the Tumour infiltrating lymphocytes in triple-negative breast cancer(TNBC), human epidermal growth factor receptor 2(HER2) - enriched breast cancer and hormone-receptor breast cancer(HRBC) [3,4]. Recent studies have gone to show the relevance of tumour infiltrating lymphocytes(TILs) as a potential biomarker for breast cancer [5] and have showcased the importance of neoadjuvant chemotherapy(NAC) employed in breast cancer patients to achieve pathological complete response(pCR) and increase the breast conservation rate thereby improving the surgical outcome and reducing the risk of distant metastases.

TILs have proven to be a predictor of outcome and recurrence in colorectal cancer [6] and ovarian cancer [7] and tumour infiltrating lymphocyte therapy has been also employed in cancers with bleak prognoses such as metastatic melanoma [8]. Though TILs have been employed as a biomarker for the aforementioned cancers, over the years there has been a shifting emphasis on its use in invasive breast carcinoma and therefore by characterising the breast carcinoma by subtype and immune environment it will help in providing insight into effective therapy and a larger population of breast cancer patients will benefit from the targeted immune therapy.

## 2 Material and methods

The given study was registered and certified under the Indian Council of Medical Research (ICMR) Short Term Studentship-2019 (STS-2019) (Ref. No. 2019–02057). Institutional Ethics Committee approval was obtained from The Institutional Ethics Committee of Krishna Institute of Medical Sciences, Karad (Reference number - KIMSDU/IEC/02/2019) following protocol approval (can be made available from the corresponding author on reasonable request). The study was reported following STROCSS 2021 guidelines [9]. The observational study was conducted on Archival Sections. The minimum number of subjects to be included was obtained according to *Asano Y.* et al. [4].

$$n=\frac{\left(p_{H}q_{H}+p_{L}q_{L}\right)*{\left(Z_{1-\alpha /2}+Z_{1-\beta }\right)}^{2}}{{\left(p_{H}-p_{L}\right)}^{2}}$$

Where:

pH: Is the frequency of High TIL in pathological complete response HRBC (17.4%)
qH: Is the frequency of High TIL in non-pathological complete response HRBC (82.6%)
pL: Is the frequency of Low TIL in pathological complete response HRBC (29.8%)
qL: Is the frequency of Low TIL in non-pathological complete response HRBC (70.2%)

Thereby, 180 patients who completed neoadjuvant chemotherapy were included for this study whose post-surgically resected tumour specimens were sectioned and stained using routine Haematoxylin and Eosin techniques.

FNAC/Core Needle Biopsy confirmed breast cancer patients who had undergone neoadjuvant chemotherapy followed by surgical resection of the tumour were included while patients having inflammatory or benign lesions of the breast were excluded.

### 2.1 Procedure

Pathological complete response was defined as there being no invasive and no in situ residuals in breast and nodes according to Gunter von Minckwitz et al. [10]. Pathological complete response for each subtype was determined by the means of the above definition.

The evaluation and analysis of the Intratumoural and Intrastromal Infiltrating Lymphocytes were done according to the guidelines put forward by Salgado et al. [11].

The method used for evaluation of Stromal Tumour infiltrating Lymphocytes:

1. The stromal area for TIL evaluation was identified. TILs immediately adjacent to the tumour border were included and TILs closely related to the tumour cells, invasive margin, and the residual tumour bed were excluded. Areas of crush artefacts, necrosis, inflammation, and hyalinisation were not considered.
2. Inflammatory infiltrate was determined. Only mononuclear cells - lymphocytes and plasma cells were included. Polymorphonuclear leukocytes were excluded.
3. The percentage of stromal TILs was assessed. It was measured semiquantitatively as a continuous variable. The average TIL from the different microscopic fields was taken and expressed in percentage. Stromal TIL % was the area occupied by mononuclear inflammatory cells over the total stromal area.
4. Depending on the percentage, the evaluation was done as follows:
  High grade TIL: 50–90% stromal TILs.
  Low Grade TIL: 0–10% stromal TILs.
  (Intermediate grade TIL: 20–40% stromal TILs. For the intermediate group different areas were evaluated at higher magnification.)

The method used for evaluation of Intratumoural infiltrating Lymphocytes:

1. The tumour area for TIL evaluation was identified. TILs closely related to the tumour cells were included and TILs closely related to the invasive margin were excluded. Areas of crush artefacts, necrosis, inflammation, and hyalinisation were not considered.
2. Inflammatory infiltrate was determined. Only mononuclear cells - lymphocytes and plasma cells will be included. Polymorphonuclear leukocytes were excluded.
3. The percentage of intratumoral TILs was assessed. It was measured semiquantitatively as a continuous variable. The average TIL from the different microscopic fields was taken and expressed in percentage. Intratumoral TIL % was the area occupied by mononuclear inflammatory cells over the total tumour area.
4. Depending on the percentage, the evaluation was done as follows:
  High grade TIL: 50–90% tumour TILs.
  Low Grade TIL: 0–10% tumour TILs.
  (Intermediate grade TIL: 20–40% tumour TILs. For the intermediate group different areas were evaluated at higher magnification.)
  For the above, 2 slides from the same tumour were evaluated and the average value of the 2 slides was taken as the final value.
  Depending on the evaluation of TIL in stroma and tumour cells, a comparison between the clinicopathological and pathological complete response (pCR) was made.

### 2.2 Statistical analysis

Data was collected manually on a pro forma and entered into a secure spreadsheet and categorised according to subtype and features. Thereafter, univariate analyses were conducted by chi-squared tests on a 2x2 contingency table. Data was analysed on InStat software where p values < 0.05 were considered significant with a 95% confidence interval. Multivariate analyses were carried out on SPSS software by performing logistical regression.

### 2.3 Observations and results

Out of the 180 breast cancer patients taken in this study 179 patients were female and 1 patient was male. 55 patients (i.e. 30.56%) out of 180 displayed pathological complete resolution (pCR). Further, the intratumoral and intrastromal TIL count was calculated for all 180 patients.

Intratumoral group: There were a total of 180 patients out of which 66 patients (i.e. 36.67%) were categorised into the high intratumoral TIL group (since the intratumoral count was between 50% and 90%) and 114 patients (i.e. 63.33%) were categorised into the low intratumoral TIL group (since the intratumoral count was between 0% and 10%).

Intrastromal group: There were a total of 180 patients out of which 105 patients (i.e. 58.33%) were categorised into the high intrastromal TIL group (since the intrastromal count was found to be between 50% and 90%) and 75 patients (i.e. 41.67%) were categorised into the low intrastromal TIL group (since the intrastromal count was found to be between 0% and 10%).

The IT and IS TIL scores were then compared to the clinicopathological features and the following was found:

On comparison of Intratumoural TIL with the clinicopathological features, the following was established at a 95% Confidence Interval as found in Table 1 - There was a very strong association of Age (p = 0.0045) with the Intratumoural TIL count followed by a significant association of Menopause (p = 0.0156). An extremely significant linkage was found with the HRBC subtype (p = 0.0008) and a notable relation with the TNBC (p = 0.0121) subtype. A significant association was found between the pathological complete response (p = 0.0335) and the Intratumoural TIL count. No correlation was found between any other clinicopathological feature or breast cancer subtype.

**Table 1:** Univariate analysis of Intratumoural TIL with Clinicopathological features in patients with Breast Cancer.

Sr. No.	Parameter		Intratumoural TIL		chi -square	p - value	Odds Ratio	Cofidence Interval
			High (n = 66)	Low (n = 114)				
1	Age at Operation	≤52 years	47 (71.21%)	55 (48.25%)	8.068	0.0045	2.654	1.389–5.070
		>52 years	19 (28.79%)	59 (51.75%)				
2	Menopause (n = 179; One Patient is male)	Premenopausal	37 (56.06%)	40 (35.40%)	6.438	0.0112	2.328	1.252–4.331
		Postmenopausal	29 (43.94%)	73 (64.60%)				
3	Tumour Size	≤4.5 cm	42 (63.64%)	74 (63.91%)	0.0001160	0.9914	0.9459	0.5027–1.780
		>4.5 cm	24 (36.36%)	40 (35.09%)				
4	Lymph Node Status	Not Involved	29 (43.94%)	57 (50.00%)	0.3964	0.5289	0.7838	0.4262–1.442
		Involved	37 (56.06%)	57 (50.00%)				
5	Periodal Spill	Negative	54 (81.82%)	90 (78.95%)	0.07327	0.7866	1.2000	0.5551–2.594
		Positive	12 (18.18%)	24 (21.05%)				
6	Tumour Emboli	Negative	44 (66.67%)	74 (64.91%)	0.005768	0.9395	1.081	0.5698–2.051
		Positive	22 (33.33%)	40 (35.09%)				
7	Subtype HRBC	HRBC	17 (25.76%)	60 (52.63%)	11.259	0.0008	0.3122	0.1609–0.6060
		Non-HRBC	49 (74.24%)	54 (47.36%)				
8	Subtype TNBC	TNBC	36 (54.55%)	39 (34.21%)	6.299	0.0121	2.308	1.241–4.291
		Non-TNBC	30 (45.45%)	75 (65.79%)				
9	Subtype HER2BC	HER2BC	10 (15.15%)	15 (13.16%)	0.02223	0.8815	1.179	0.4963–2.799
		Non- HER2BC	56 (84.85%)	99 (86.84%)				
10	Subtype TPBC	TPBC	02 (03.03%)	00 (00.00%)	1.280	0.2579	8.876	0.4193–187.7
		Non-TPBC	64 (96.97%)	114 (100.00%)				
11	Subtype Luminal B	Luminal B	01 (01.52%)	00 (00.00%)	0.07698	0.7814	5.244	0.2104–130.07
		Non-Luminal B	65 (98.48%)	114 (100.00%)				
12	Pathological Complete Response	pCR	27 (40.91%)	28 (24.56%)	4.522	0.0335	2.126	1.110–4.074
		Non-pCR	39 (59.09%)	86 (75.44%)				

On comparison of Intrastromal TIL with the clinicopathological features, it was recognised that at the 95% Confidence Interval as shown in Table 2 - There was a significant association of age (p = 0.0147) with the intrastromal TIL count followed by a highly significant association found with the HRBC subtype (p < 0.0001), TNBC subtype (p < 0.0001) and HER2BC subtype (p = 0.0025). A noteworthy association was found between the pathological complete response (p < 0.0001) and the Intrastromal TIL count. No correlation was found between any other clinicopathological feature or breast cancer subtype.

**Table 2:** Univariate analysis of Intrastromal TIL with Clinicopathological features in patients with Breast Cancer.

Sr. No.	Parameter		Stromal TIL		chi - square	p - value	Odds Ratio	Cofidence Interval
			High (n = 105)	Low (n = 75)				
1	Age at Operation	≤52 years	68 (64.76%)	34 (45.33%)	5.957	0.0147	2.216	1.209–4.062
		>52 years	37 (35.24%)	41 (54.67%)				
2	Menopause (n = 179; One Patient is male)	Premenopausal	50 (47.62%)	27 (36.49%)	1.764	0.1841	1.582	0.8607–2.910
		Postmenopausal	55 (52.38%)	47 (63.51%)				
3	Tumour Size	≤4.5 cm	65 (61.90%)	51 (68.00%)	0.4683	0.4938	0.7647	0.4093–1.429
		>4.5 cm	40 (38.10%)	24 (32.00%)				
4	Lymph Node Status	Not Involved	48 (45.71%)	38 (50.67%)	0.2545	0.6139	0.8199	0.4528–1.485
		Involved	57 (54.29%)	37 (49.33%)				
5	Periodal Spill	Negative	83 (79.05%)	61 (81.33%)	0.03571	0.8501	0.8659	0.4101–1.828
		Positive	22 (20.95%)	14 (18.67%)				
6	Tumour Emboli	Negative	71 (67.62%)	47 (62.67%)	0.2812	0.5959	1.244	0.6683–2.316
		Positive	34 (32.38%)	28 (37.33%)				
7	Subtype HRBC	HRBC	23 (21.90%)	54 (72.00%)	42.829	<0.0001	0.1091	0.05503–0.2162
		Non-HRBC	82 (78.10%)	21 (28%)				
8	Subtype TNBC	TNBC	57 (54.29%)	18 (24.00%)	15.288	<0.0001	3.760	1.954–7.236
		Non-TNBC	48 (45.71%)	57 (76.00%)				
9	Subtype HER2BC	HER2BC	22 (20.95%)	03 (04.00%)	9.143	0.0025	6.361	1.828–22.142
		Non- HER2BC	83 (79.05%)	72 (96.00%)				
10	Subtype TPBC	TPBC	02 (01.90%)	00 (00.00%)	0.2311	0.6307	3.647	0.1725–77.136
		Non-TPBC	103 (98.10%)	75 (100.00%)				
11	Subtype Luminal B	Luminal B	01 (00.95%)	00 (00.00%)	0.7183	0.3967	2.167	0.08703–53.981
		Non-Luminal B	104 (99.05%)	75 (100.00%)				
12	Pathological Complete Response	pCR	48 (45.71%)	7 (09.33%)	25.602	<0.0001	8.180	3.435–19.483
		Non-pCR	57 (54.29%)	68 (90.67%)				

**Fig. 1:**
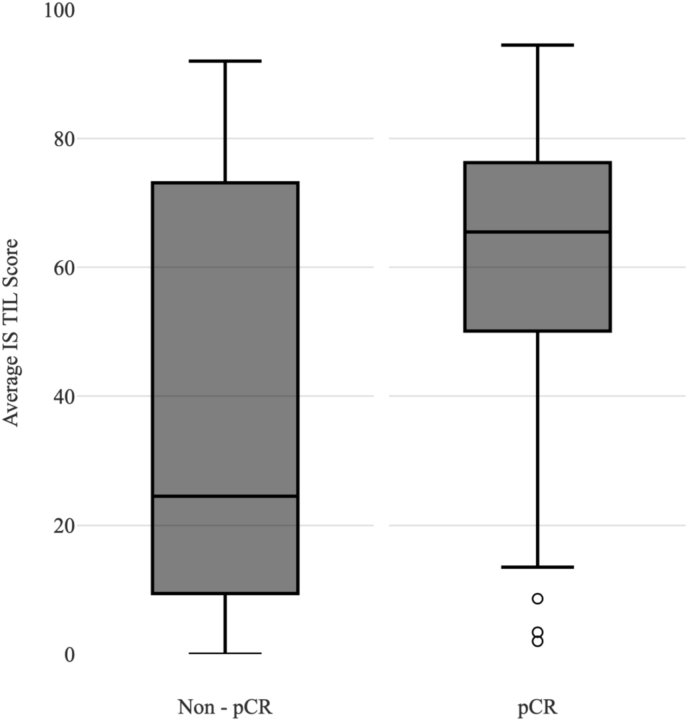
Intrastromal TIL score association with pCR.

As depicted in Fig. 1, it is evident that the Intrastromal TIL count has a higher concentration and is skewed upwards in pCR patients with a high TIL score while the plot is observed to be evenly distributed in the Non-pCR patients. The median is also found to be higher in pCR patients compared to Non-pCR patients and the interquartile range is much larger in Non-pCR.

**Fig. 2:**
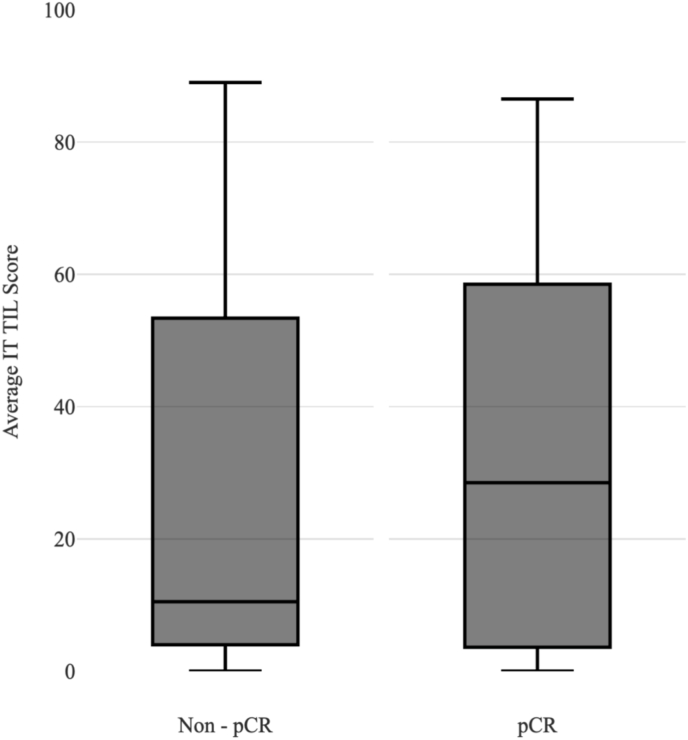
Intrastromal TIL score association with pCR.

Through Fig. 2, it is clear that the Intratumoural TIL count is evenly distributed in both the pCR and Non-pCR patients. The median is slightly higher in pCR patients compared to Non-pCR with both in the intermediate and low TIL score range and the interquartile ranges for pCR and Non-pCR were similar.

On comparison between the Intratumoural and Intrastromal TIL plots, it is apparent that the pCR patients have a higher median and smaller interquartile range focused in the high Intrastromal TIL score area as opposed to the Intratumoural TILs which have a lower median and larger interquartile range distributed in the lower score range area of the graph. This therefore could be the reason for the stronger association of high Intrastromal TIL scores and pCR in comparison to the high Intratumoural TIL score and pCR rate.

For Multivariate Analysis, One male patient was excluded. Therefore the sample size considered was n = 179.

### 2.4 In the intratumoral group

Logistic regression was performed to predict Intratumoural TIL by introducing 7 variables as independent parameters as found in Table 3, Table 4, Table 5. Backward regression revealed that four (age at operation, pathological complete response, lymph node involvement and menopause) of those parameters highly contributed to predicting 68.2% of the total cases correctly with sensitivity of 93.0% and specificity of 24.6% when the cut off probability was 0.5.

**Table 3:** Parameter coding reference for multivariate analysis of IT TIL.

Sr. No.	Parameter		Frequency	Parameter Coding
1	Menopause	Premenopausal	77	0.000
		Postmenopausal	102	1.000
2	Pathological Complete Response	pCR	55	0.000
		Non-pCR	102	1.000
3	Lymph Node Involvement	Not Involved	85	0.000
		Involved	94	1.000
4	Age at Operation	≤52 years	101	0.000
		>52 years	78	1.000

**Table 4:** Classification ability of the model is given in Table 3.

Observed		Predicted		
		IT TIL		Percentage Correct (%)
		High	Low	
IT TIL	High	16	49	24.6
	Low	8	106	93.0
Overall Percentage				68.2

**Table 5:** Logistic Regression model to predict High and Low IT TIL.

Sr. No.	Parameter	B	S.E.	Wald	dF	Sig.	Exp(B)	95% C.I. for Exp(B)	
								Lower	Upper
1	Age at Operation	0.700	0.403	3.009	1	0.083	2.013	0.913	4.438
2	Pathological Complete Response	0.790	0.355	4.950	1	0.026	2.204	1.099	4.420
3	Lymph Node Involvement	−0.411	0.339	1.464	1	0.226	0.663	0.341	1.290
4	Menopause	0.465	0.393	1.403	1	0.236	1.592	0.737	3.437
Constant		−0.259	0.475	0.296	1	0.586	0.772		

### 2.5 In the intrastromal group

Logistic regression was performed to predict Intrastromal TIL by introducing 7 variables as independent parameters as depicted in Table 6, Table 7, Table 8. Backward regression revealed that four (age at operation, pathological complete response, lymph node involvement and tumour emboli) of those parameters highly contributed to predicting 71.5% of the total cases correctly with sensitivity of 71.6% and specificity of 71.4% when the cut off probability was 0.5.

**Table 6:** Parameter coding reference for multivariate analysis of IS TIL.

Sr. No.	Parameter		Frequency	Parameter Coding
1	Lymph Node Involvement	Not Involved	85	0.000
		Involved	94	1.000
2	Pathological Complete Response	pCR	55	0.000
		Non-pCR	102	1.000
3	Tumour Emboli	Negative	117	0.000
		Positive	62	1.000
4	Age at Operation	≤52 years	101	0.000
		>52 years	78	1.000

**Table 7:** Classification ability of the model given in Table 6.

Observed		Predicted		
		IS TIL		Percentage Correct (%)
		High	Low	
IS TIL	High	75	30	71.4
	Low	21	53	71.6
Overall Percentage				71.5

**Table 8:** Logistic Regression model to predict High and Low IS TIL.

Sr. No.	Parameter	B	S.E.	Wald	dF	Sig.	Exp(B)	95% C.I. for Exp(B)	
								Lower	Upper
1	Age at Operation	0.946	0.345	7.495	1	0.006	2.575	1.308	5.067
2	Pathological Complete Response	2.260	0.467	23.453	1	0.000	9.585	3.840	23.924
3	Tumour Emboli	0.207	0.364	0.323	1	0.570	1.230	0.603	2.509
4	Lymph Node Involvement	−0.575	0.358	2.580	1	0.108	0.563	0.279	1.135
Constant		−2.251	0.476	22.317	1	0.000	0.105		

## 3 Discussion

TILs have been found to play an important role in the microenvironment of the tumour and can help predict the behaviour of the tumour [5]. Not only restricting the prediction to the tumour environment, but the neoadjuvant chemotherapy employed also contributes to improving the surgical outcome to achieve pathological complete resolution. Multiple studies have been carried out on Intrastromal TILs and their significance in measuring the disease-free survival rates and the likelihood of recurrence [3,12,13], while the use of Intratumoural TILs to forecast the response is seldom performed.

Guidelines put forward by Salgado et al. [11] throw light upon recent studies having found stromal TILs to be of a higher calibre and more reproducible when compared to its counterpart. The main reason for this is that Intratumoral TILs are scarce and the process of delineating them from tumour cells on H&E-stained slides is rather tedious and the growth pattern of these lymphocytes could be disturbed by the nest of tumour cells. Therefore, this study was solely focused on comparing and contrasting the above two to come to a consensus on their relative effectiveness.

On comparing TIL with the Clinicopathological features, the Intratumoural TILs proved to be highly significant with age (p = 0.0045) where 59 out of 78 members with age greater than 52 years were found to garner a Low IT TIL score. This can be pinned down to the fact that as age increases there is a steady decline in the immunogenicity of an individual [14] and therefore the lymphocytic response is reduced in the microenvironment of the tumour and this low response can be indicative of a bad prognosis.

On the contrary, IS TIL revealed a similar significance with age (p = 0.0147) where a higher concentration of stromal TIL was found in patients with an age lesser than 52 years. This comes down to the fact that immunogenicity in the tumour environment is higher in lower-age patients; therefore, the higher the immunogenicity, the better the prognosis.

The IT TILs had a strong correlation with menopause (p = 0.0156) where 72 out of 101 postmenopausal women exhibited a lower IT TIL score. Gameiro et al. [15] studied the prognosis of breast cancer in postmenopausal women and found that after menopause there is a decrease in CD4 T lymphocytes and B lymphocytes. This shows that the immune reaction is lower in postmenopausal women, leading to the low score obtained within the tumour.

IT TILs had a slight association with the pCR (p = 0.0335) whereas Intrastromal TILs had a significantly large association with pCR (p < 0.0001) which is dependent on the lymphocytic response.

As seen in the above results, TILs are closely related to the prediction of neoadjuvant chemotherapy with the pathological complete response of breast cancer and can serve as a stratification factor in clinical trials. They provide vital information not only on the response to chemotherapy but through this study have opened up avenues for further research on their relations with clinicopathological features mainly age at operation and menopausal status.

## 4 Conclusion

The cases taken for this study resided in rural areas and comprised of low socio-economic status members who face substantial hurdles in receiving preventive health care services and therefore report to doctors at a much later stage where the tumour size has exponentially increased and the tumour reveals rather high grades which indicate bad prognosis. The tertiary care centre where this study was conducted caters to the needs of such patients by providing them with the facility of neoadjuvant chemotherapy to both reduce the large tumour size and infiltration to achieve pathological complete resolution.

Therefore, the evaluation and analysis of TILs should be made standardised and reproducible to be made universally available to all patients with breast cancer. TILs can be used as a potential biomarker for utilisation as a prognostic tool in breast cancer, the evaluation for which does not require any additional material and can be done by using routine Haematoxylin and Eosin stained tumour sections. This, if employed in rural areas where necessary equipment for predicting the outcome of breast cancer might not be available or might be costly, will prove to be a cost-effective and reliable method for the prediction of treatment response and immunogenicity of the tumour.

## Provenance and peer review

Not commissioned, externally peer-reviewed.

## Ethical approval

The study was approved by the Institutional Ethics Committee of Krishna Institute of Medical Sciences, Karad (Reference number - KIMSDU/IEC/02/2019).

## Source of funding

Indian Council of Medical Research - Short Term Studentship (ICMR-STS) - 2019 Program funded research project [Ref. No. 2019–02057].

## Author contribution

RR was the major contributor in writing the entire article and analysing the results. SRK was the guide and imparted knowledge related to how to analyse the TILs and was the overseer of the analysis of the project. SJB was the Onco-surgeon in charge whose post-surgically resected breast tumour specimens were employed for the project. SVK helped in multivariate analysis. NJP facilitated the IHC marking for the project. AG was the oncologist who oversaw the neoadjuvant chemotherapy for the patients. All authors read and approved the final manuscript.

## Consent for publication

Not applicable.

## Guarantor

**Dr. Rahul Rangan,** 502-Peregrine, Raheja Woods, Kalyani Nagar, Pune - 411006, Maharashtra, India, **Tel:** +91 9404730188, **Email:** rahul.rangan98@yahoo.co.in.

## Trail registry number

Name of the registry: Indian Council of Medical Research (ICMR).

Unique Identifying number or registration ID: 2019-02057.

## Availability of data and materials

The datasets used and/or analysed during the current study are available from the corresponding author on reasonable request.

## Declaration of competing interest

The authors have declared no conflicts of interest.

## Abbreviations

TNBC: Triple-negative breast cancer
TPBC: Triple positive breast cancer
HER2: human epidermal growth factor receptor 2
HRBC: Hormone-receptor breast cancer
TILs: Tumour infiltrating lymphocytes
NAC: Neoadjuvant chemotherapy
pCR: Pathological complete response
